# Engeletin alleviates depressive‐like behaviours by modulating microglial polarization via the LCN2/CXCL10 signalling pathway

**DOI:** 10.1111/jcmm.18285

**Published:** 2024-04-10

**Authors:** Jie Zhang, Zheng Song, Yanchao Huo, Guangqiang Li, Liming Lu, Chuanmei Wei, Shuping Zhang, Xinfu Gao, Xingyue Jiang, Yangyang Xu

**Affiliations:** ^1^ Department of Radiology Binzhou Medical University Hospital Binzhou Shandong P. R. China; ^2^ Department of Pharmacy Binzhou Medical University Hospital Binzhou Shandong P. R. China; ^3^ College of Basic Medicine Binzhou Medical University Yantai Shandong P.R. China

**Keywords:** chronic social defeat stress, CXCL10, engeletin, inflammatory, LCN2, magnetic resonance imaging, microglial polarization

## Abstract

Microglial polarization and associated inflammatory activity are the key mediators of depression pathogenesis. The natural *Smilax glabra rhizomilax* derivative engeletin has been reported to exhibit robust anti‐inflammatory activity, but no studies to date have examined the mechanisms through which it can treat depressive symptoms. We showed that treatment for 21 days with engeletin significantly alleviated depressive‐like behaviours in chronic stress social defeat stress (CSDS) model mice. T1‐weighted imaging (T1WI), T2‐weighted imaging (T2WI) imaging revealed no significant differences between groups, but the bilateral prefrontal cortex of CSDS mice exhibited significant increases in apparent diffusion coefficient and T2 values relative to normal control mice, with a corresponding reduction in fractional anisotropy, while engeletin reversed all of these changes. CSDS resulted in higher levels of IL‐1β, IL‐6, and TNF‐a production, enhanced microglial activation, and greater M1 polarization with a concomitant decrease in M2 polarization in the mPFC, whereas engeletin treatment effectively abrogated these CSDS‐related pathological changes. Engeletin was further found to suppress the LCN2/C‐X‐C motif chemokine ligand 10 (CXCL10) signalling axis such that adeno‐associated virus‐induced LCN2 overexpression ablated the antidepressant effects of engeletin and reversed its beneficial effects on the M1/M2 polarization of microglia. In conclusion, engeletin can alleviate CSDS‐induced depressive‐like behaviours by regulating the LCN2/CXCL10 pathway and thereby altering the polarization of microglia. These data suggest that the antidepressant effects of engeletin are correlated with the polarization of microglia, highlighting a potential avenue for future design of antidepressant strategies that specifically target the microglia.

## 1 INTRODUCTION

Major depressive disorder (MDD) affects approximately one out of every six people over the course of their lifetime, and remains a leading cause of morbidity and disability throughout the world. While the cellular mechanisms, molecular pathways, and neural circuits that are correlated with the incidence of depression are increasingly well understood, the precise biological factors that give rise to this disease remain incompletely understood, hampering efforts to design new, effective antidepressants.^1^, ^2^

Neuroinflammatory processes are increasingly thought to contribute to the incidence and progression of depressive disorders.^3^, ^4^ Patients suffering from clinical depression exhibit significantly elevated levels of inflammatory cytokines including IL‐6, IL‐1β and TNF‐α.^5^ Consistent with these changes, rodent models of depression established through chronic unpredictable mild stress or lipopolysaccharide (LPS) exposure exhibit significant increases in the levels of these inflammatory mediators.^6^, ^7^ Such neuroinflammation is characterized by the activation of resident immune effector cells within the central nervous system (CNS), including the microglia.^8^ Microglia serve as essential regulators of microenvironmental homeostasis through their ability to detect pathogens and eliminate them via phagocytosis, in addition to secreting inflammatory chemokines and cytokines that support tissue repair and nervous system development.^9^, ^10^ The activation of microglia and the consequent induction of neuroinflammation have been tied to the pathogenesis of MDDs. Broadly speaking, activated microglia are classified into proinflammatory M1 cells and anti‐inflammatory regenerative M2 cells.^11^, ^12^ Higher levels of M1 microglia polarization and activity are associated with phagocytic and inflammatory activity, and an elevated M1/M2 ratio is related to impaired neurological function and depression in rat.^13^, ^14^ Efforts aimed at suppressing aberrant microglial activation and/or restoring the appropriate homeostatic balance between M1 and M2 microglia thus represent promising new antidepressant strategies.

**FIGURE 1:**
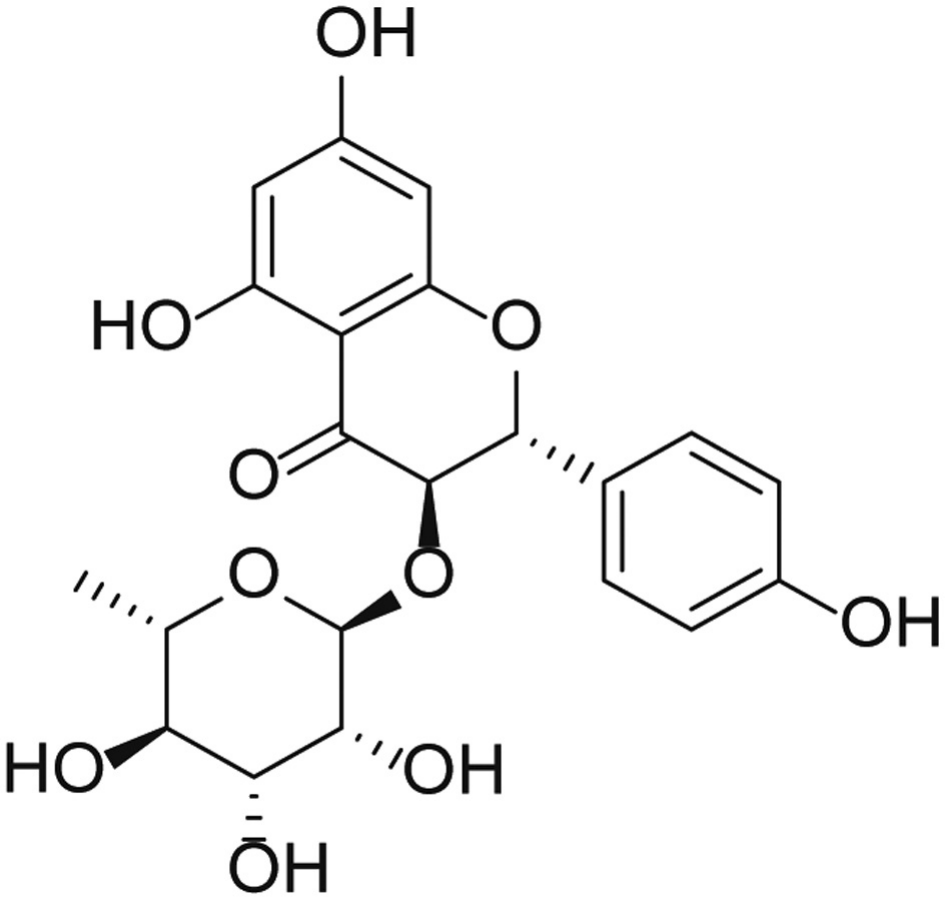
The chemical structure of engeletin.

Engeletin (dihydrokaempferol 3‐rhamnoside) is a natural flavanonol glycoside derived from *Smilax glabra rhizomilax* extracts (Figure 1). Engeletin has previously been shown to exhibit antitumorigenic, antioxidant and anti‐inflammatory properties.^15^, ^16^ In addition, it has been shown to exert neuroprotective activity, suppressing the production of reactive oxygen species and mitigating the severity of ischemia/reperfusion injury and Alzheimer's disease.^17^, ^18^ Preliminary research conducted by our group further revealed that engeletin can alleviate depressive‐like behaviours induced by chronic restraint stress through its ability to modulate the brain‐derived neurotrophic factor (BDNF)/TrkB/mTORC1 signalling axis, thereby enhancing synaptic plasticity.^19^ However, no research to date has evaluated the effects of engeletin on the regulation of the M1/M2 polarization of microglia.

To address this gap in knowledge, this study was developed to explore the therapeutic effects of engeletin on depressive‐like behaviours and to clarify the underlying antidepressant mechanisms in an effort to establish new approaches to controlling this devastating disease.

## 2 MATERIALS AND METHODS

### 2.1 Drugs and animals

Engeletin (C_21_H_22_O_10_), was provided by SenBeiJia Biological Technology (Jiangsu, China). Male C57BL/6J mice (weight 20–24 g) were purchased from Jinan Pengyue Experimental Animal Center (licence number: SCXK20190003). Mice were maintained at a constant room temperature of 22 ± 3°C, with a humidity of 50% ± 15%, and were kept at a 12/12 h light/dark cycle.

### 2.2 Chronic social defeat stress (CSDS) and social interaction (SI) test

Briefly, each adult male C57BL/6 experimental mice was exposed to 5–10 min of physical aggression by a male CD‐1 mice. At the completion of the session, C57BL/6J experimental and CD‐1 mice were housed overnight in a two‐compartment rat cage and separated by a transparent divider to provide sensory, but not physical, contact. The procedure was repeated for a total of 10 consecutive days, in which C57BL/6J experimental mice faced a new aggressor every day. After 10 days of chronic stress social defeat stress (CSDS), sensitive mice were screened out using the social interaction (SI) test for subsequent experiments, and were grouped into single cages according to the social interaction ratio.

For the social interaction (SI) test, we measured the time spent in the interaction zone (IZ) during the first (target absent) and second (target present) trials.^20^ The time of mice spent in the IZ surrounding the plastic box was recorded and analysed.

### 2.3 Behavioural measurements

#### 2.3.1 Sucrose preference test (SPT)

To observe the anhedonia‐like behaviour, the SPT was performed as described.^21^ The process is as follows: (1) 1% sucrose solution and (2) tap water. SPT was performed for 6 h, and the liquid consumption was assessed by subtracting the bottle weights. The consumption was calculated as follows: The sucrose preference rate = (1% sucrose solution intake)/[(1% sucrose solution intake) + (water intake)].

#### 2.3.2 Forced swimming test (FST)

The FST was performed in a clear glass cylinder (height 25 cm, diameter 10 cm) filled with 10 cm of water (25 ± 1°C). All mice were forced to swim for 6 min, and during the last 4 min, the immobility time was recorded.

#### 2.3.3 Tail suspension test (TST)

TST is a test to evaluate despair or depressive‐like behaviour. Briefly, adhesive tape was stuck to the tail's tip about 1 cm below to suspend mice for 6 min, 50 cm above the floor. The time during which mice remained immobile over a period of 4 min was recorded.

#### 2.3.4 Open field test (OFT)

OFT was used to assess the motor abilities of mice. Mice were placed in a plexiglass arena (50 cm × 50 cm × 50 cm) and were allowed to explore the open field freely for 10 min. The total travelled distance was scored.

### 2.4 Magnetic resonance imaging (MRI)

After a series of behavioural experiments, MRI images were captured using a 3.0 T horizontal magnet (Skyra, Siemens, Munich, Germany) with a 25‐mm‐diameter gradient coil (four channels for mice, Chen Guang Medical Co. Ltd, Shanghai, China). Axial T1‐weighted imaging (T1WI), axial T2‐weighted imaging (T2WI), T2‐mapping, and diffusion tensor imaging (DTI) were acquired. Imaging parameters are as follows: T1‐mprage: repetition time (TR) = 2200 ms, echo time (TE) = 3.86 ms, matrix size (MTX) = 224 × 224, slice thickness (ST) = 0.5 mm, field‐of‐view (FOV) = 60 mm × 52 mm; T2WI: TR = 4800 ms, TE = 102 ms, MTX = 3320 × 320, ST = 1.5 mm, FOV = 63 mm × 63 mm; DTI: TR = 3000 ms, TE = 67 ms, MTX = 142 × 142, ST = 1.5 mm, FOV = 108 mm × 82 mm; T2‐mapping: TR = 1360 ms, TE = 16.1–69 ms, MTX = 384 × 384, ST = 2.0 mm, FOV = 103 mm × 103 mm.

### 2.5 RNA‐Seq and data analysis

The high‐throughput RNA sequencing analysis for this study was provided by a commercial service (Biotech Biotechnology Inc, Shanghai, China). First, total RNA was extracted from the medial prefrontal cortex (mPFC) tissue of three groups of mice (three samples per group): CON group, CSDS group and engeletin group, respectively. The FastQC software was applied to perform quality control analysis of the read sequences from RNA‐seq data. Trimmomatic is used for quality cutting to obtain relatively accurate and effective data. The Q20 and P80 were used as thresholds to remove lower quality read sequences. And then we used HISAT2 to align the RNA‐seq reads to reference genome sequences of mouse (GRCm38) that obtained from NCBI database. According to the reference transcripts from NCBI database, we used salmon software to calculate the expression level of transcripts for each sample, and next obtained DEG by R package ‘DESeq2’. Genes with |log2FoldChange| is >1 (|log2FC| >1, difference >2 fold), with *q*‐value <0.05, were chosen for further analysis by setting parameters on the Illumina NovaSeq6000 platform.

### 2.6 Viral constructs and mPFC viral infusion

For local overexpression of LCN2 in mPFC, a virus packed with a non‐fusion protein expression vector, adeno‐associated virus (AAV2/9)‐CMV‐MCS‐3flag‐T2A‐ZsGreen made by Hanbio Biotechnology Co. Ltd. (1.6 × 10^12^ vg/mL, Shanghai, China) was injection in C57BL/6J mice. AAV‐CMV‐ZsGreen (1.4 × 10^12^ vg/mL, Shanghai, China) was used as the control vector. Mice were anaesthetised with sodium pentobarbital (80 mg/kg, i.p.) before surgery. Subsequently, 2 μL of AAV vectors were stereotaxically microinjected into the bilateral mPFC (AP, 2.2 mm; ML, ±0.3 mm; DV, −2.4 mm) at 0.1 μL/min. The needle was slowly withdrawn after 10 min.

### 2.7 Immunohistochemical staining

Immunohistochemistry was performed as previously described.^22^ The primary antibodies included anti‐IBA1 (ab178846, Rabbit, 1:200). The sections were observed using a confocal laser scanning microscope (model FV1000, Olympus).

### 2.8 RNA extraction and real‐time PCR

Gene expression levels were measured by RT‐qPCR as described in our previous publication using GAPDH as the internal control. Primer sequences in detail are shown in Table 1.

**TABLE 1:** Primer sequences used for RT‐PCR analysis.

Gene	Forward (5′‐3′)	Reverse (5′‐3′)
IL‐10	GGCAGAGAACCATGGCCCAGAA	AATCGA TGACAGCGCCTCAGCC
IL‐1β	AAATGCCACCTTTTGACAGTG	GAGTGATACTGCCTGCCTGA
IL‐6	GACAAAGCCAGAGTCCTTCAGA	GAGCATTGGAAATTGGGGTAGG
TNF‐α	TGCCTCAGCCTCTTCTCATT	GGGCTTGTCACTCGAGTTTT
LCN2	CCCCATCTCTGCTCA CTGTC	TTTTTCTGGACCGCATTG
CXCL10	TCTGAGTGGGACTCAAGGGAT	TTGTGGCAATGATCTCAACATG
GAPDH	GCAGTGGCAAAGTGGAGATTG	TGCAGGATGCATTGCTGACA

### 2.9 Western blottings

The primary antibodies included rabbit anti‐ IBA1 (ab178846, 1:1000), rabbit anti‐BDNF (ab108319, 1:1000), rabbit anti‐iNOS (ab178945, 1:1000), rabbit anti‐CD86 (ab239075, 1:1000), and rabbit anti‐CD206 (#24595, 1:1000), rabbit anti‐Arg1 (PA5‐29645, 1:5000), mice anti‐LCN2 (ab125075, 1:1000), rabbit anti‐C‐X‐C motif chemokine ligand 10 (CXCL10) (ab306587, 1:1000) and GAPDH (AF0006, 1:1000). Western blotting images were captured using Super Signal West Pico Chemiluminescent Substrate (Thermo Fisher Scientific Inc.). The acquired data were normalized with the relative GAPDH density.

### 2.10 Statistical analysis

Data were expressed as the mean ± standard error of the mean (SEM) and analysed using GraphPad Prism 9.4.1. Statistical analysis was performed using one‐way or two‐way ANOVA test followed by Bonferroni post hoc testing. Differences were considered statistically significant when *p* < 0.05.

## 3 RESULTS

### 3.1 Engeletin treatment alleviates depressive‐like behaviours in CSDS model mice

**FIGURE 2:**
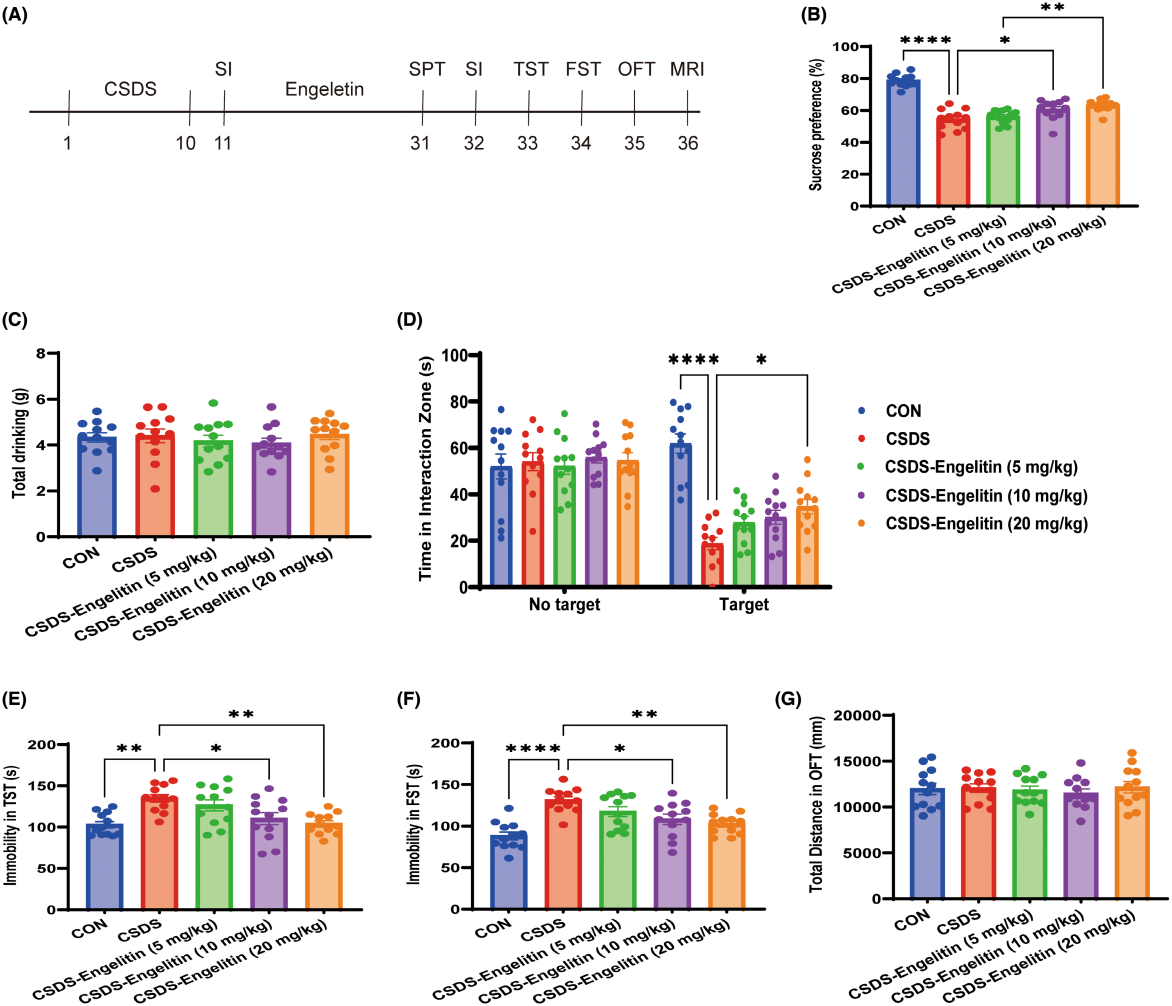
Engeletin alleviates CSDS‐induced depressive‐like behaviours. (A) An overview of the relative timing of CSDS modelling, engeletin treatment, and behavioural testing (SPT, SIT, TST, FST and OFT), which was performed at the end of the study for all mice. (B–G) The effects of engeletin on SPT (B, C), SIT (D), TST (E), FST (F) and open field test (OPT) (G) test performance. Results are means ± SEM. (*n* = 12). *p < 0.05, **p < 0.01, ****p < 0.0001, one‐ or two‐way ANOVAs with Bonferroni post hoc testing. CSDS, chronic stress social defeat stress; FST, forced swimming test; SIT, social interaction test; TST, tail suspension test.

As they exhibit robust validity as predictors of antidepressant‐like activity, the FST and TST are behavioural tests that are widely used when assessing possible approaches to combatting depressive symptoms.^3^ Engeletin has previously been reported to induce a dose‐dependent (5, 10, 20 mg/kg) reduction in immobility time in these two testing paradigms. Whether engeletin exhibits robust antidepressant activity in the CSDS model of depression; however, has yet to be established. To test this possibility, engeletin was intragastrically administered (5, 10, 20 mg/kg) for 21 days following CSDS modelling (Figure 2A). Under these conditions, engeletin was associated with a significant improvement in percentage reduction in sucrose consumption (Figure 2B), whereas it had a relatively minimal impact on water drinking (Figure 2C). The repeated administration of engeletin also restored CSDS‐associated reductions in social interactions (Figure 2D), while also fully reversing the CSDS‐related increases in murine immobility observed in the TST and FST (Figure 2E,F). Neither stress‐related modelling nor engeletin administration; however, had any impact on the locomotor behaviour of mice in the OFT (Figure 2G).

### 3.2 The impact of engeletin on MRI scan results in CSDS model mice

**FIGURE 3:**
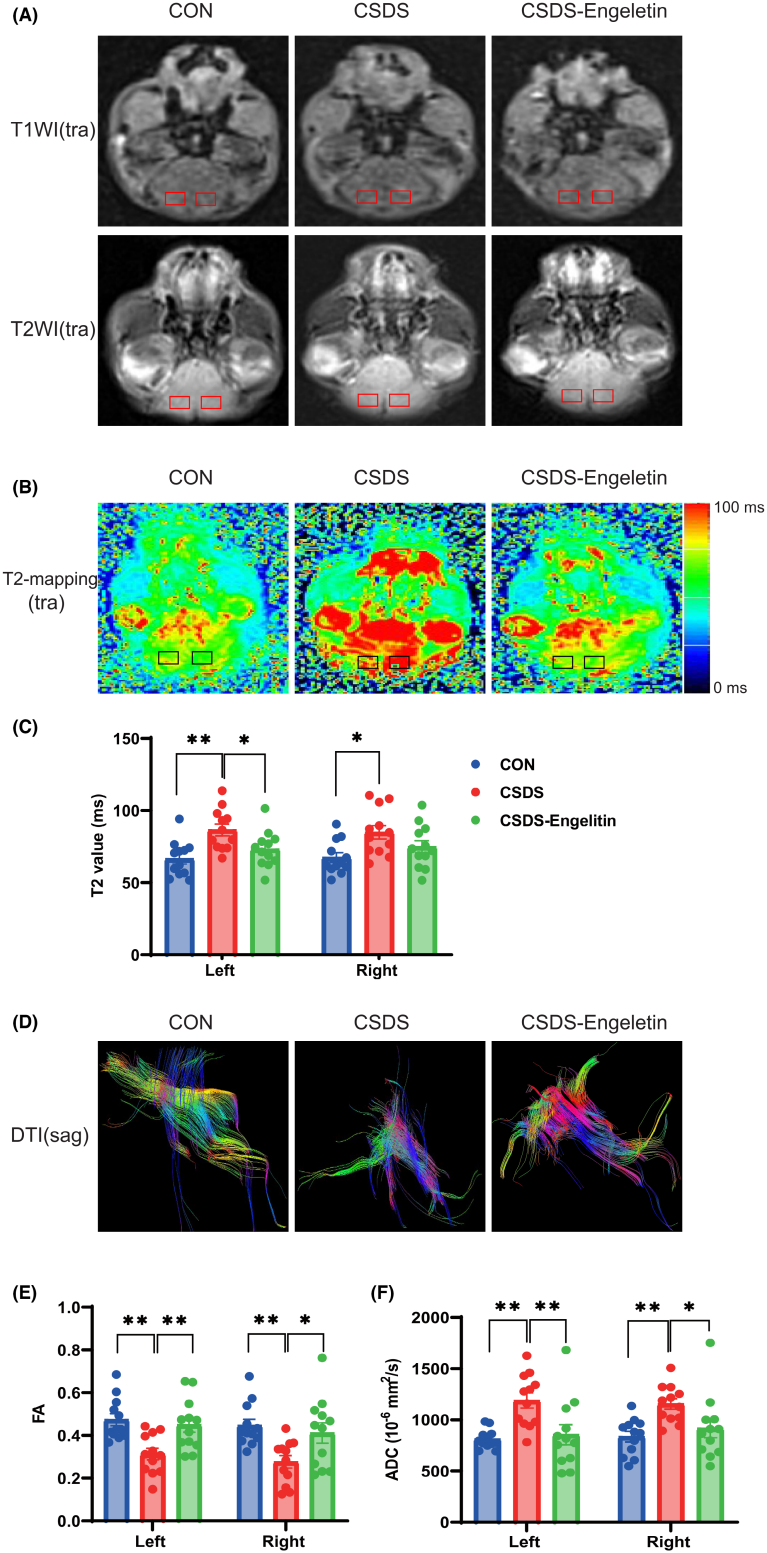
The impact of engeletin on multimodel magnetic resonance imaging (MRI) results in CSDS model mice. (A) Representative T1‐weighted imaging (T1WI) and T2‐weighted imaging (T2WI) images from the CON, CSDS, and engeletin groups. (B, C) Representative T2 mapping images and corresponding quantification. (C, D) Representative diffusion tensor imaging (DTI) images. (E, F) DTI index quantification (FA, ADC) in the bilateral prefrontal cortex. Results are means ± SEM (*n* = 12). *p < 0.05, **p < 0.01, one‐way ANOVAs with Bonferroni post hoc testing. ADC, apparent diffusion coefficient. CSDS, chronic stress social defeat stress.

Neuroimaging strategies can detect MDD‐related functional and structural changes in many neurological circuits.^23^ While no differences between the control, CSDS, and engeletin‐treated groups were detected via T1WI or T2WI (Figure 3A), CSDS modelling was associated with a significant increase in the T2 value of the bilateral prefrontal cortex (Figure 3B). This suggests the presence of inflammation and oedema in the prefrontal lobe in this model of depression, while engeletin was able to reverse these changes (Figure 3C). Fractional anisotropy (FA) values were also significantly decreased in the bilateral prefrontal cortex of these CSDS model mice, with a marked increase in the apparent diffusion coefficient (ADC), with engeletin administration reversing both of these pathological changes (Figure 3D–F).

### 3.3 Engeletin suppresses CSDS‐induced inflammatory cytokine production within the mPFC

**FIGURE 4:**
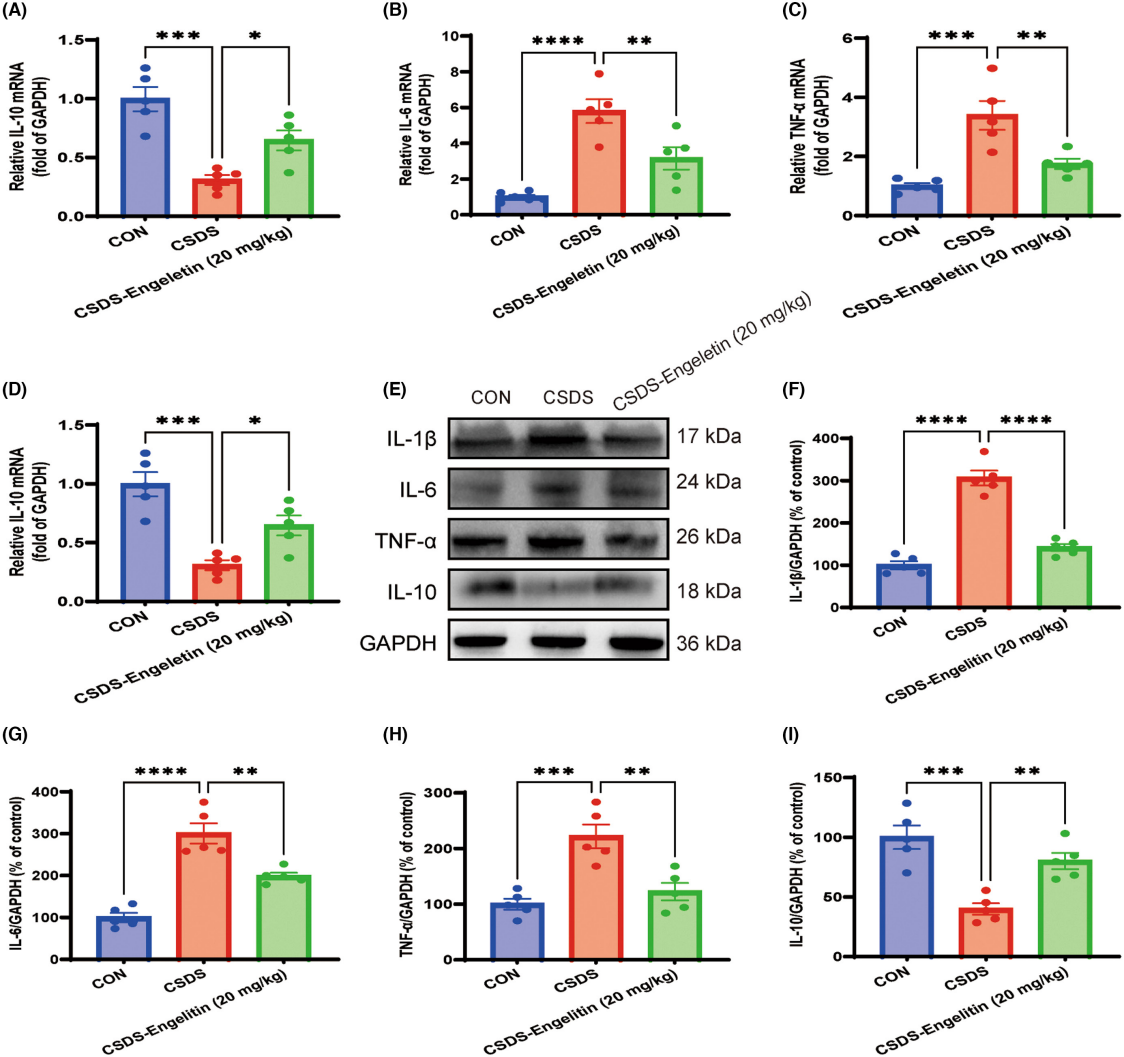
Engeletin suppresses inflammatory cytokine production within the medial prefrontal cortex (mPFC) in CSDS model mice. (A–D) quantitative real‐time PCR (qRT‐PCR) analyses of IL‐1β, IL‐6, TNF‐α and IL‐10 expression. (E–I) Western immunoblotting analyses of IL‐1β, IL‐6, TNF‐α and IL‐10 expression. Results are means ± SEM (*n* = 5). *p < 0.05, **p < 0.01, ***p < 0.001, ****p < 0.0001, one‐way ANOVAs with Bonferroni post hoc testing. CSDS, chronic stress social defeat stress.

Accumulating evidence suggests that the mPFC is a key brain region in the regulation of behaviours and emotions. Additionally, more and more studies have found that neuroinflammation is of great significance in the development of depression. Therefore, to gain additional insight into the immunomodulatory potential of engeletin, quantitative real‐time PCR (qRT‐PCR) was next used to assess changes in inflammatory cytokine expression in mPFC. As shown in Figure 4A–D, CSDS mice showed a significant increase in pro‐inflammatory IL‐1β, IL‐6 and TNF‐α, diminution in anti‐inflammatory IL‐10 levels. Treatment with engeletin (20 mg/kg) markedly attenuated the concentrations of IL‐1β, IL‐6 and TNF‐α, improved the concentrations of IL‐10. Furthermore, as shown in Figure 4E–I, western blotting similarly confirmed that engeletin was sufficient to reverse CSDS‐induced increases in pro‐inflammatory cytokine levels (IL‐1β, IL‐6 and TNF‐α) and reductions in IL‐10 levels.

### 3.4 Engeletin modules the polarization of microglia in CSDS model mice

**FIGURE 5:**
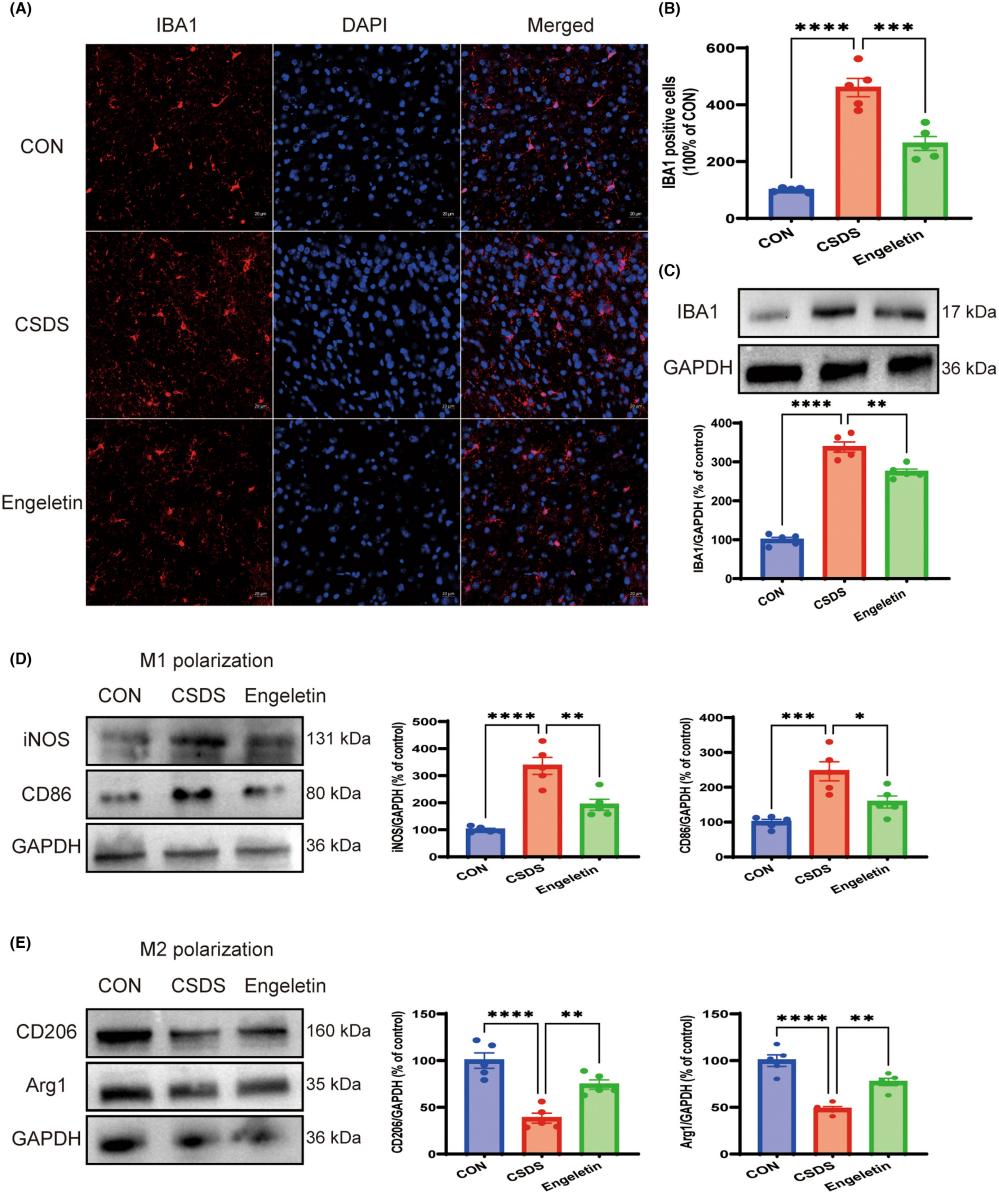
Engaletin modulates the activation and polarization of microglia within the medial prefrontal cortex (mPFC). (A, B) Representative immunofluorescent staining for IBA1 (A) and corresponding quantification. Scar bar: 20 μm. (C–E) Western blotting analyses of IBA1 (C), markers of M1 microglial polarization (CD86, iNOS) (D), and the markers of M2 microglial polarization (Arg1, CD206) (E). Results are means ± SEM (*n* = 5). *p < 0.05, ** *p* < 0.01, **** *p* < 0.0001, one‐way ANOVAs with Bonferroni post hoc testing.

To better understand how engeletin impacts microglial polarization in the context of CSDS‐driver neuroinflammatory activity, immunofluorescent staining and western blotting were next employed to evaluate the degree of M1 and M2 polarization within the mPFC of mice in these different experimental groups. Significant increases in IBA1 staining and protein levels were detected in the mPFC samples from the CSDS model group relative to the control group, while engeletin administration alleviated this change (Figure 5A–C). Engeletin additionally suppressed polarization towards the pro‐inflammatory M1 microglial phenotype, as evidenced by reductions in the expression of CD86 and iNOS, while promoting the upregulation of CD206 and Arg‐1 consistent with the induction of M2 polarization (Figure 5D,E). Overall, these data thus suggest that engeletin is capable of serving as an anti‐inflammatory mediator through its ability to modulate microglial polarization.

### 3.5 Engeletin suppresses CSDS‐induced LCN2/CXCL10 pathway signalling

**FIGURE 6:**
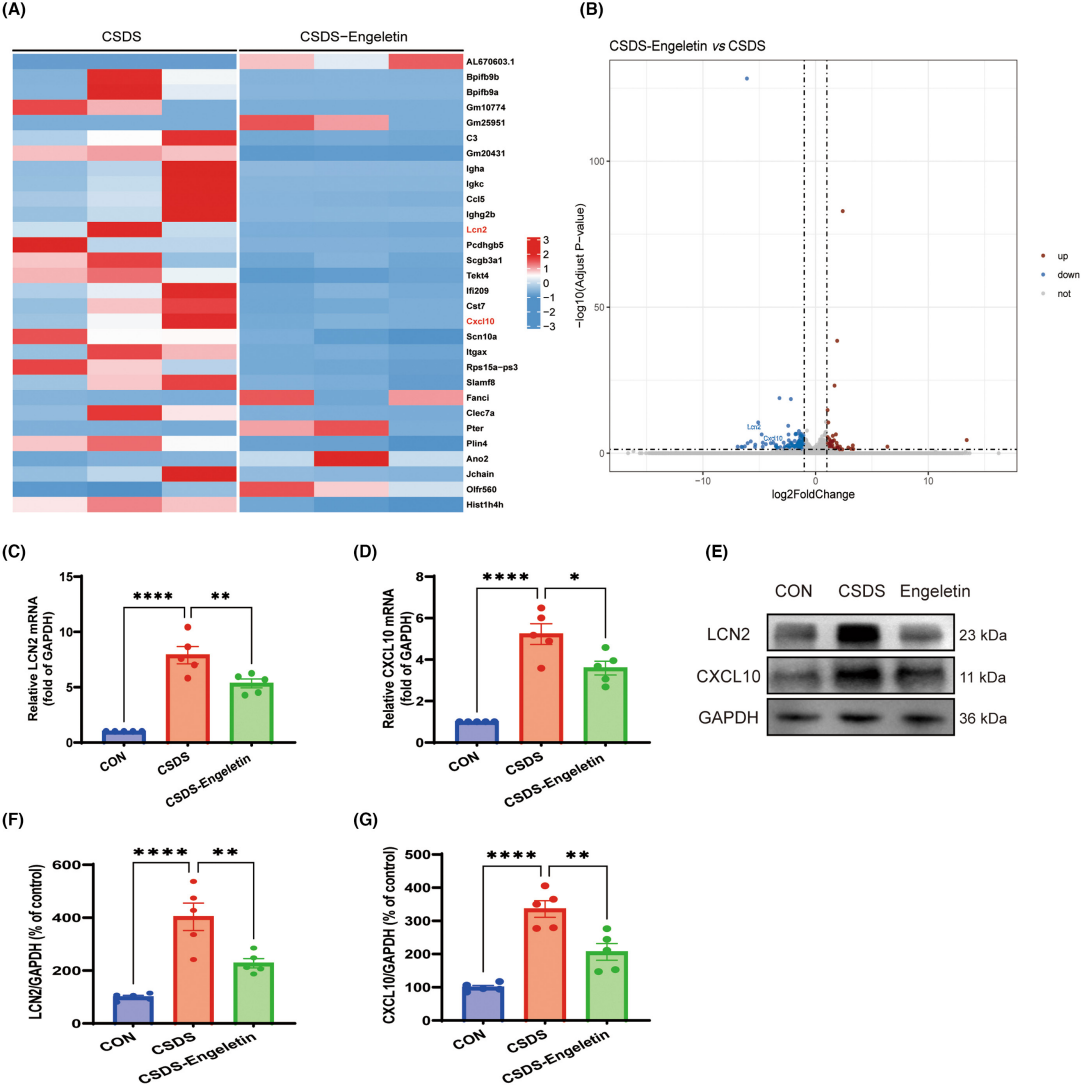
Engeletin suppresses LCN2/CXCL10 signalling activity in the medial prefrontal cortex (mPFC). (A, B) Partial heatmaps and volcano plots highlighting differences in gene expression between the CSDS and engeletin groups (|log2FC|>1, difference>2 fold), with blue and red respectively indicating downregulated and upregulated genes (*n* = 3). (C, D) quantitative real‐time PCR (qRT‐PCR) analyses of changes in LCN2 and CXCL10 expression in the CON, CSDS, and engeletin groups (*n* = 5). (E–G) Representative western blotting (E) and corresponding quantification (F, G) of LCN2 and CXCL10 expression in the mPFC (*n* = 5). Results are means ± SEM. *p < 0.05, **p < 0.01, ****p < 0.0001, one‐way ANOVAs with Bonferroni post hoc testing. CSDS, chronic stress social defeat stress.

To clarify the mechanisms whereby engeletin controls microglial polarization, mPFC samples were collected for transcriptomic sequencing from the CSDS and engeletin treatment groups. Analyses of the top 30 most significantly differentially expressed genes revealed pronounced LCN2 and CXCL10 downregulation within the mPFC following engeletin treatment (Figure 6A,B). LCN2 has been suggested to act as an M1‐amplifier in brain microglial cells since it is secreted from M1‐polarized. Furthermore, LCN2 up‐regulates the expression of CXCL10, which acts in a paracrine or autocrine manner to induce cell migration. In line with these results, significant decreases in LCN2 and CXCL10 expression following engeletin treatment were confirmed via qRT‐PCR and western blotting (Figure 6C–G).

### 3.6 Engeletin suppresses LCN2/CXCL10 signalling to alleviate depressive‐like behaviours

**FIGURE 7:**
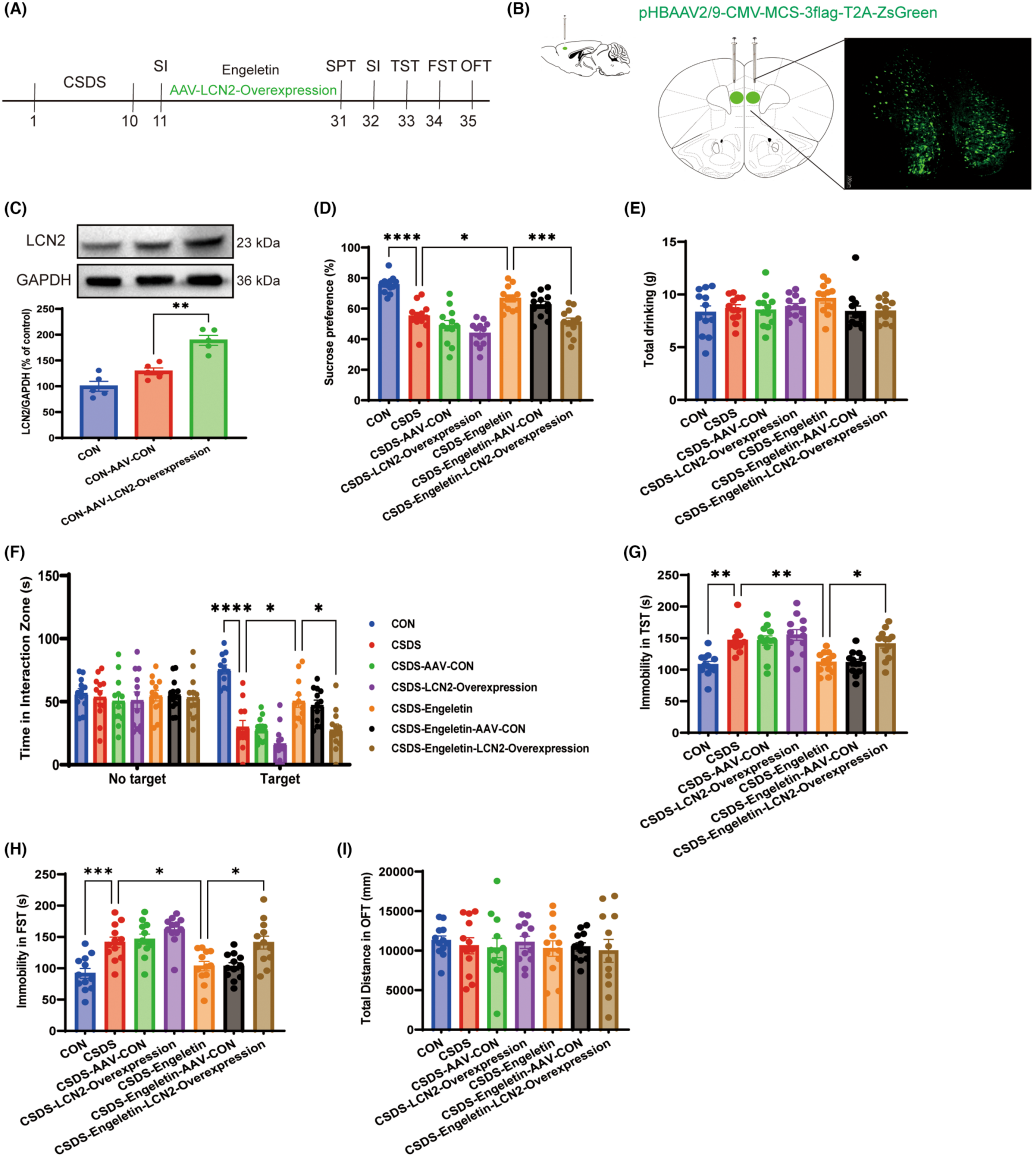
LCN2 overexpression ablates the in vivo antidepressant‐like activity of engeletin. (A) Overview of the experimental approach used to assess behavioural responses in mice following adeno‐associated virus (AAV)‐mediated LCN2 overexpression. (B) Fluorescent images of a fixed brain section expressing AAV‐LCN2‐EGFP in the medial prefrontal cortex (mPFC) on Day 14 following AAV stereotactic infusion. Scale bar: 100 μm. (C) Western blotting confirmed successful AAV‐LCN2‐EGFP overexpression (*n* = 5). (D–H) LCN2 overexpression was found to reverse the antidepressant‐like effects of engeletin on murine behaviours in the SPT (D, E), SIT (F), TST (G), and FST (H) (*n* = 12). (I) No significant differences in locomotor function were observed among groups in the OFT (*n* = 12). Results are means ± SEM. *p < 0.05, ***p < 0.001, ****p < 0.0001, one‐way ANOVAs with Bonferroni post hoc testing. FST, forced swimming test; SIT, social interaction test; TST, tail suspension test.

LCN2 was next overexpressed in the mPFC using an AAV‐LCN2‐EGFP vector in order to probe whether the antidepressant effects of engeletin are mechanistically related to LCN2/CXCL10 pathway inhibition (Figure 7A). AAV‐LCN2‐EGFP expression remained stable for 14 days following stereotaxic infusion, and overexpression was validated through both immunofluorescent imaging and western blotting (Figure 7B,C). Behavioural analyses revealed that CSDS model mice in which LCN2 was overexpressed that were treated with engeletin exhibited significant reductions in sucrose preference and social interactions (Figure 7D–F) relative to CSDS model mice administered the AAV‐CON vector and engeletin, together with increased immobility in the TST and FST assays (Figure 7G–I).

### 3.7 LCN2 overexpression abrogates the impact of engeletin on the polarization of microglia

**FIGURE 8:**
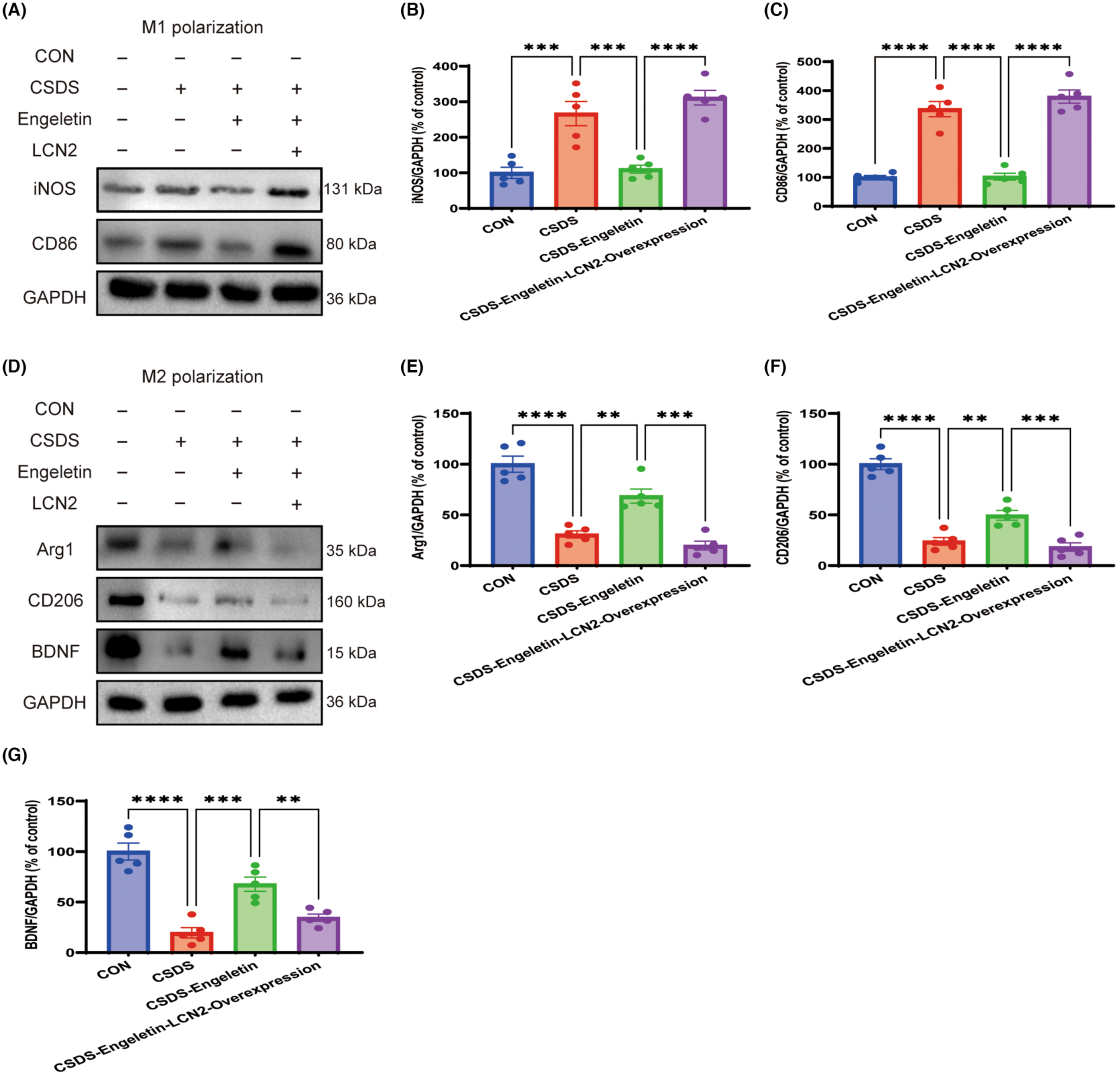
LCN2 overexpression in the medial prefrontal cortex (mPFC) reverses the engeletin‐mediated shift in the M1 and M2 polarization of microglia in CSDS model mice. (A–C) Representative western blotting (A) and corresponding quantification (B, C) demonstrating that the overexpression of LCN2 reverses engeletin‐related reductions in the M1 polarization of microglia (iNOS, CD86). (D–G) Representative western blotting (D) and corresponding quantification (E–G) demonstrating that the overexpression of LCN2 reverses the engeletin‐induced enhancement of M2 microglial polarization (CD206, Arg1 and BDNF). Results are means ± SEM. (*n* = 5). *p < 0.05, **p < 0.01, ***p < 0.001, ****p < 0.0001, one‐way ANOVAs with Bonferroni post hoc testing. BDNF, brain‐derived neurotrophic factor; CSDS, chronic stress social defeat stress.

In order to clarify the potential link between the LCN2/CXCL10 signalling axis and the impact of engeletin on the polarization of microglia within CSDS model mice, changes in microglial marker expression were assessed in the mPFC following LCN2 overexpression. These experiments revealed that overexpressing LCN2 eliminated the ability of engeletin to suppress iNOS and CD86 upregulation following CSDS modelling (Figure 8A–C), while reversing engeletin‐related increases in Arg‐1, CD206, and BDNF expression in these mice (Figure 8D–G). These results suggest that the ability of engeletin to inhibit M1 microglial polarization while favouring polarization towards an M2 phenotype in this mouse CSDS model system is at least partially dependent on LCN2/CXCL10 pathway activation.

## 4 DISCUSSION

Here, a murine model of CSDS‐induced depression was employed to explore the potential antidepressant activity of the *Smilax glabra Roxb*.‐derived flavonol glycoside engeletin. Ultimately these analyses revealed that engeletin is able to alleviate CSDS‐related depressive‐like phenotypes through its ability to suppress LCN2/CXCL10 pathway activation and thereby modulate microglial polarization.

Stress responses entail the engagement of a diverse range of physiological and behavioural responses.^24^ Exposure to chronic stress over extended periods can contribute to the incidence of MDD and other adverse outcomes. The precise biological mechanisms that give rise to MDD remain incompletely understood, and therapeutic options remain limited owing to the heterogeneous nature of this disease and the pathogenic and clinical levels.^25^, ^26^ Diagnosing MDD in a clinical setting is also highly dependent on structured interviews and subjective criteria, contributing to a greater risk of patients being misdiagnosed, thus increasing the burden of this disease.

Structural MRIs have led to the identification of several brain abnormalities in MDD patients, with these changes most commonly manifesting in grey matter areas related to decision‐making, emotional procession, and reward circuitry within the limbic system and frontal lobe.^27^, ^28^ Multiparameter MRI scans are currently regarded as the most effective approach to noninvasively analysing the pathological basis of MDD *in vivo*.^29^ T1WI and T2WI strategies enable the visualization of brain anatomy, whereas quantitative indices such as DTI measurements and T2 values can provide quantitative sensitivity when attempting to detect injury to the nervous system and gauge the efficacy of neuroprotective interventions.^30^ The T2 hyperintensity of the bilateral prefrontal cortex is associated with increases in inflammation, vascular permeabilization, myelin turnover, water content and the accumulation of by‐products of myelin and axonal breakdown.^31^, ^32^ ADC and FA values are key quantitative DTI parameters that are closely correlated with the pathogenesis of depression. These quantitative DTI and T2 parameters can also be evaluated and compared with the outcomes of behavioural and histological analyses to gain more robust insight into the incidence of depression and to explore therapeutic outcomes.^33^, ^34^ Here, ADC, FA, and T2 values for the bilateral prefrontal cortex were significantly altered in CSDS model mice, while engeletin administration reversed these changes and alleviated depressive‐like behavioural phenotypes. Overall these findings conclusively demonstrate that T2 and DTI measures can offer value as biomarkers of depression that can gauge disease progression and enable the rigorous assessment of interventional strategies.

Chronic stress is one of the most important causes of depression, accompanied by neuroinflammation and prefrontal cortex injuries. Indeed, pharmacological strategies to curb the pathological effects of persistent neuroinflammation are of interest for many disorders of the CNS, which was beneficial to exert a protective effect to regulate biological behaviours.^14^, ^35^

Furthermore, the role of microglia in the neuroinflammatory response that occurs in the development of depression has been demonstrated.^36^ Microglia displays distinct morphologies as either amoeboid or ramified cells, indicating distinct functional states.^37^, ^38^ After stimulation by external pathogenic factors, such as LPS and chronic stress, microglia transform into the amoeboid‐state and secrete a large number of inflammatory mediators and cytotoxic molecules, leading to damage in peripheral nerve cells.^39^ The use of anti‐inflammatory drugs to suppress microglia‐mediated inflammation has been shown to alleviate depressive symptoms, and a growing body of evidence suggests that depressive‐like phenotypes can arise owing to the imbalance between pro‐ and anti‐inflammatory cytokine production.^38^, ^40^ Here, engeletin was found to promote the upregulation of anti‐inflammatory IL‐10 within the mPFC in CSDS model mice while suppressing the expression of pro‐inflammatory IL‐1β, IL‐6 and TNF‐α. As peripheral cytokines are generally limited in their ability to cross the blood–brain barrier, microglia serve as a main source of these inflammatory mediators within the CNS, promoting neuroinflammation that is central to the pathogenesis of CSDS‐associated depressive‐like behaviours.

Microglia are mesoderm‐derived cells present within neurological tissues that are central to depression‐related pathways and to the incidence of neuroinflammation.^10^ While microglial polarization has been found to correlate with depressive behaviour, the specific mechanisms underlying this correlation and their therapeutic relevance remain to be fully clarified. Microglia that exhibit M1 and M2 polarization profiles secrete differing levels of pro‐ and anti‐inflammatory cytokines, in addition to expressing different functional and morphological markers.^41^ M1 microglia, which generally express iNOS and CD86, produce high concentrations of inflammatory cytokines such as IL‐6, TNF‐α, IFN‐γ and IL‐1β, whereas M2 microglia express Arg1 and CD206, and secrete anti‐inflammatory factors including IL‐10, IL‐4 and TGF‐β.^9^ M2 microglial polarization has been demonstrated to have beneficial effects following chronic stress in the context of neurological disease.^42^ Here, treatment with engeletin resulted in M2 microglia activation and the suppression of M1 polarization, in turn increasing IL‐10 and TGF‐β secretion while suppressing IL‐1β, TNF‐α and IL‐6 production within the mPFC. These results thus suggested that engeletin is capable of treating CSDS through the tuning of the microglial balance of M1/M2 polarization and the alleviation of neuroinflammation, potentially providing a novel avenue for the treatment of neurological disease.

LCN2 has increasingly been shown to play a central role in the incidence of neuroinflammatory pathology in the CNS.^43^ Also referred to as NGAL or 24p3, LCN2 is a lipocalin family member that binds to a range of hydrophobic molecules, interacts with particular cell surface receptors, and controls concentrations of iron within cells, thereby influencing an array of pathways.^44^, ^45^ While the expression of LCN2 at baseline is relatively limited, it can be rapidly upregulated whereupon it serves as a regulator of viability, migratory activity, innate immunity, and tissue morphology. Multiple studies have identified links between LCN2, depression and behavior.^46^ For example, mice exposed to a 6 h restraint model of stress exhibited a 7‐fold increase in hippocampal LCN2 expression. Restraint stress also reportedly induces an increase in amygdalar LCN2 expression, with such upregulation primarily taking place within neurons and being functionally linked to increases in immature neuroplastic spines consistent with fear memory formation.^47^, ^48^ Stress‐naïve Lcn2‐knockout rodents also resent with an increase in spine density in the amygdalar basolateral complex as compared to wild‐type control animals.^49^ Naude et al.^50^ found that plasma LCN2 levels in patients with depression were significantly increased relative to non‐depressed controls, and plasma LCN2 levels were also reportedly higher in patients suffering from recurrent depression as compared to first‐episode depression.

Chemokines play an essential role in guiding the movement of particular cell populations to specific physiological sites. In addition to their importance in the maintenance of systemic homeostasis, these chemotactic cytokines can also contribute to pathological changes within the CNS, particularly in the context of development, injury, synaptic transmission and disease‐related neuroinflammatory activity.^51^ LCN2 was recently identified as a promoter of chemokine expression within the CNS, with neurons, endothelial cells, astrocytes and microglia all serving as potential cellular producers of chemokines.^46^ CXCL10 secretion induced by LCN2 has been reported to induce microglial, astrocytic and neuronal migration through mechanisms at least partially regulated by JAK2/STAT3 and IKK/NF‐κB pathways.^52^ Here, chronic stress was associated with significant LCN2/CXCL10 axis activation, whereas the administration of engeletin markedly suppressed LCN2 and CXCL10 expression within the mPFC in a manner that was reversed by LCN2 overexpression in the mPFC of these engeletin‐treated CSDS model mice.

**FIGURE 9:**
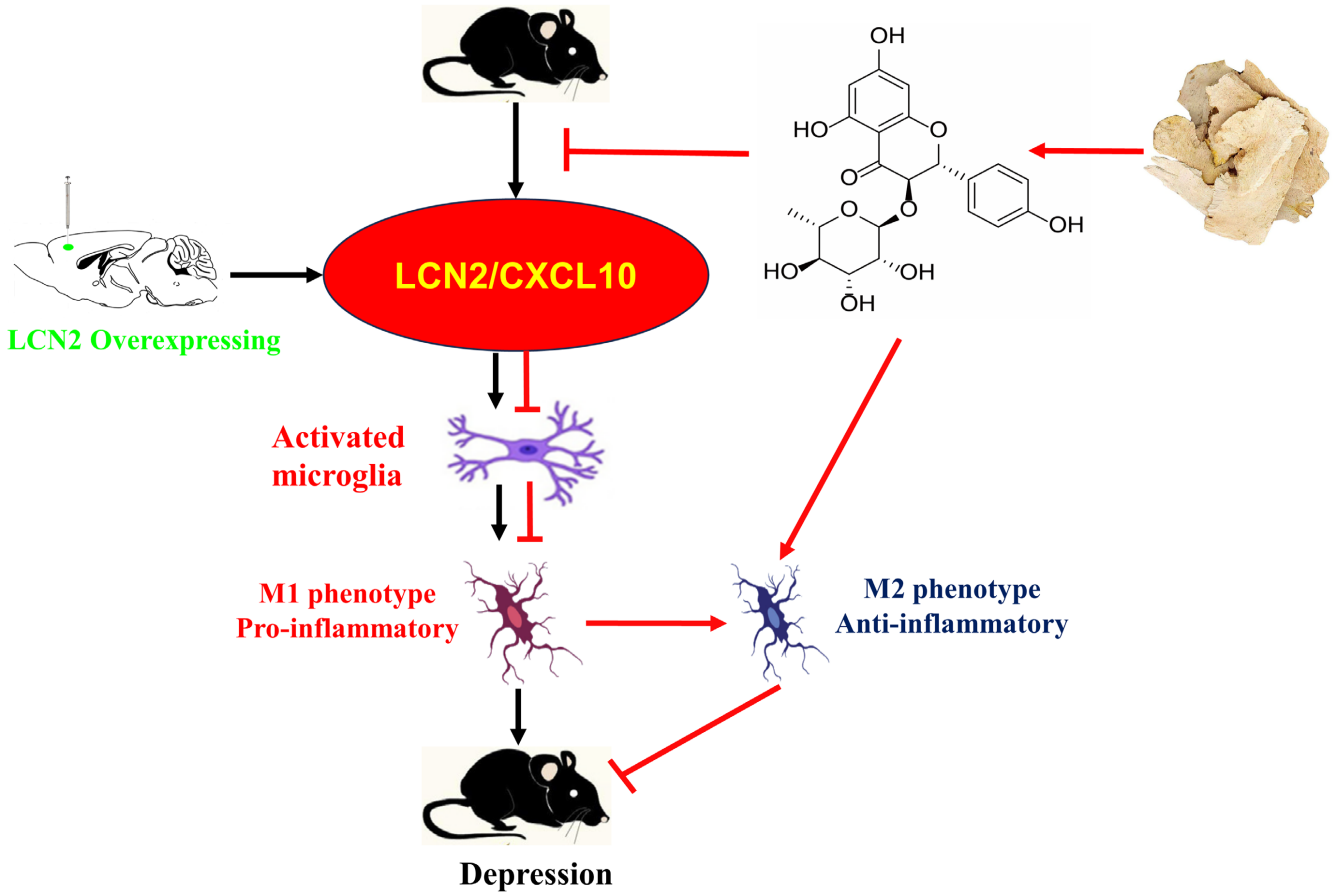
Schematic model of the mechanism by which engeletin suppresses CSDS‐induced depressive‐like behaviours by modulating microglial polarization via the LCN2/CXCL10 signalling pathway. CSDS, chronic stress social defeat stress.

LCN2 was significantly downregulated in apoptosis‐resistant microglia, and subsequent research in which LCN2 was knocked down or overexpressed revealed that it serves as a vital mediator of apoptotic sensitization and the amoeboid transformation of activated microglia.^53^, ^54^ The precise physiological importance of LCN2 as a regulator of the M1/M2 polarization of microglia; however, remains poorly understood. Here, the antidepressant‐like effects of engeletin were primarily found to be attributable to its ability to modulate LCN2/CXCL10 pathway signalling and microglial polarization. The overexpression of LCN2 was sufficient to reverse the antidepressant‐like benefits of engeletin in CSDS model mice while reducing the expression of M2‐associated proteins (Figure 9). These data provide further support for the utility of LCN2 as a diagnostic biomarker of depression that can be assessed in combination with a range of other inflammatory markers, growth factors, and metabolic or endocrine changes when assessing the incidence of depressive symptoms. It is worth noting that we only studied LCN2 as a key target for Engeletin to regulate microglial polarization. We will construct AAVs that overexpress or silence CXCL10 to explore the mechanism of CXCL10 in chronic stress and the antidepressant effect of engeletin.

## 5 CONCLUSION

Together, these data demonstrate that chronic stress is associated with LCN2 upregulation, whereas engeletin treatment is capable of mitigating inflammation‐associated damage and promoting the M2 polarization of M1 microglia through the suppression of LCN2/CXCL10 signalling activity. Of course, future studies need to further confirm the microglia polarization process by various means, such as flow cytometry for microglia sorting. In addition, cellular experiments are still needed here to complement the refinement of the molecular mechanism by which engeletin regulates microglia polarization.

## AUTHOR CONTRIBUTIONS

**Jie Zhang:** Data curation (equal); formal analysis (equal); project administration (equal); resources (equal); software (equal); writing – original draft (equal). **Zheng Song:** Conceptualization (equal); data curation (equal); funding acquisition (equal); investigation (equal); project administration (equal); writing – original draft (equal). **Yanchao Huo:** Data curation (equal); methodology (equal); project administration (equal); resources (equal); supervision (equal); writing – original draft (equal). **Guangqiang Li:** Project administration (equal); software (equal); supervision (equal); validation (equal); visualization (equal). **Liming Lu:** Data curation (equal); formal analysis (equal); investigation (equal); methodology (equal); project administration (equal); supervision (equal). **Chuanmei Wei:** Data curation (equal); formal analysis (equal); project administration (equal); supervision (equal); validation (equal); visualization (equal). **Shuping Zhang:** Formal analysis (equal); funding acquisition (equal); methodology (equal); resources (equal); supervision (equal); writing – review and editing (equal). **Xinfu Gao:** Data curation (equal); formal analysis (equal); funding acquisition (equal); investigation (equal); project administration (equal); resources (equal); visualization (equal). **Xingyue Jiang:** Funding acquisition (equal); software (equal); validation (equal); visualization (equal); writing – review and editing (equal). **Yangyang Xu:** Conceptualization (equal); data curation (equal); formal analysis (equal); funding acquisition (equal); resources (equal); writing – review and editing (equal).

## FUNDING INFORMATION

The National Natural Science Foundation of China (Grant No: 31570352 and 31170321), Project of Shandong Medical and Health Science and Technology Development Plan (Grant No: 2017WS153 and 202209010908), Project of Shandong Traditional Chinese Medicine Technology (Grant No: Q‐2023011). and Binzhou Medical University Science and Technology Program (Grant No: BY2016KJ11 and BY2020KJ43). We would like to thank all the reviewers who participated in the review and MJ Editor (www.mjeditor.com) for its linguistic assistance during the preparation of this manuscript.

## CONFLICT OF INTEREST STATEMENT

The authors declare no competing financial interests.

## DECLARATIONS

All experimental procedures in this study were conducted in accordance with the National Institutes of Health Guidelines for Care and Use of Laboratory Animals and all animal protocols were approved by the Laboratory Animals Care and Use Committee of Binzhou Medical University Hospital (20230206‐61).

## Data Availability

The data sets used and/or analysed during the current study are available from the corresponding author on reasonable request.
